# Indications, prognosis, and complications of de Novo implantable cardioverter defibrillators in patients with and without type 2 diabetes: a nationwide registry-based cohort study

**DOI:** 10.1186/s12933-025-03044-5

**Published:** 2025-12-31

**Authors:** Yiran Zhou, Elina Rautio, Per Näsman, Soffia Gudbjörnsdottir, Fredrik Gadler, Lars Rydén, Tigist Wodaje, Linda G. Mellbin

**Affiliations:** 1 Department of Medicine, Karolinska Institute, Solna, Stockholm, Sweden; 2 Heart, Vascular and Neuro Theme, Karolinska University Hospital, Stockholm, Sweden; 3 Center for Safety Research, KTH Royal Institute of Technology, Stockholm, Sweden; 4 Department of Molecular and Clinical Medicine, University of Gothenburg, Gothenburg, Sweden; 5 Department of Medicine, Karolinska Institute, Huddinge, Stockholm, Sweden

**Keywords:** Type 2 diabetes, Ventricular arrhythmia, Implantable cardioverter defibrillators, Registry-based cohort studies

## Abstract

**Background:**

Patients with type 2 diabetes have an increased risk of tachyarrhythmias and more frequently require implantable cardioverter defibrillators (ICD) than those without diabetes (No-DM). This study aims to investigate whether there is a difference in the indication, prognosis and complication rates for ICD-implantation between patients with and without type 2 diabetes in different ICD prevention groups.

**Research design and methods:**

This Swedish retrospective cohort study included patients with de novo ICDs implanted between 2010 and 2021. Data from six national registries were analyzed to compare type 2 diabetes and No-DM patients regarding indications, complications, and outcomes (major adverse cardiovascular events [MACE], all-cause mortality). Subgroup analyses compared type 2 diabetes and No-DM by primary (PP) or secondary prevention (SP) ICD indication, and within the type 2 diabetes and No-DM groups (PP vs. SP).

**Results:**

The study cohort consisted of 12,885 patients, including 2,843 with type 2 diabetes. Patients with diabetes had a mean age of 67.9 years and 85.4% were male, compared with 62.1 years and 78.1% among No-DM patients (both *p* < 0.0001). PP was more frequent in patients with type 2 diabetes (62.7%) than No-DM (54.4%, *p* < 0.0001). Ischemic heart disease was the most common etiology in both patients with/without type 2 diabetes (47.7% vs. 32.6%, *p* < 0.0001). Non-ischemic etiologies were more common in No-DM patients, e.g. dilated cardiomyopathy (15.3% vs. 17.5%, *p* = 0.007). Type 2 diabetes patients had a higher adjusted risk of all-cause mortality (Hazard ratio 1.95 [95% CI: 1.81–2.11]) and MACE (1.87 [1.71–2.05]), with a more pronounced risk in SP than PP. Infection rates were comparable between patients with type 2 diabetes and No-DM (1.1% vs. 1.3%).

**Conclusions:**

Patients with type 2 diabetes more often received ICDs for PP and ischemic indications than No-DM patients and had a worse prognosis despite similar one-year infection risk. This likely reflects greater comorbidity burden and diabetes-specific factors, indicating the need for tailored risk management strategies beyond device implantation in patients with type 2 diabetes.

**Graphical Abstract:**

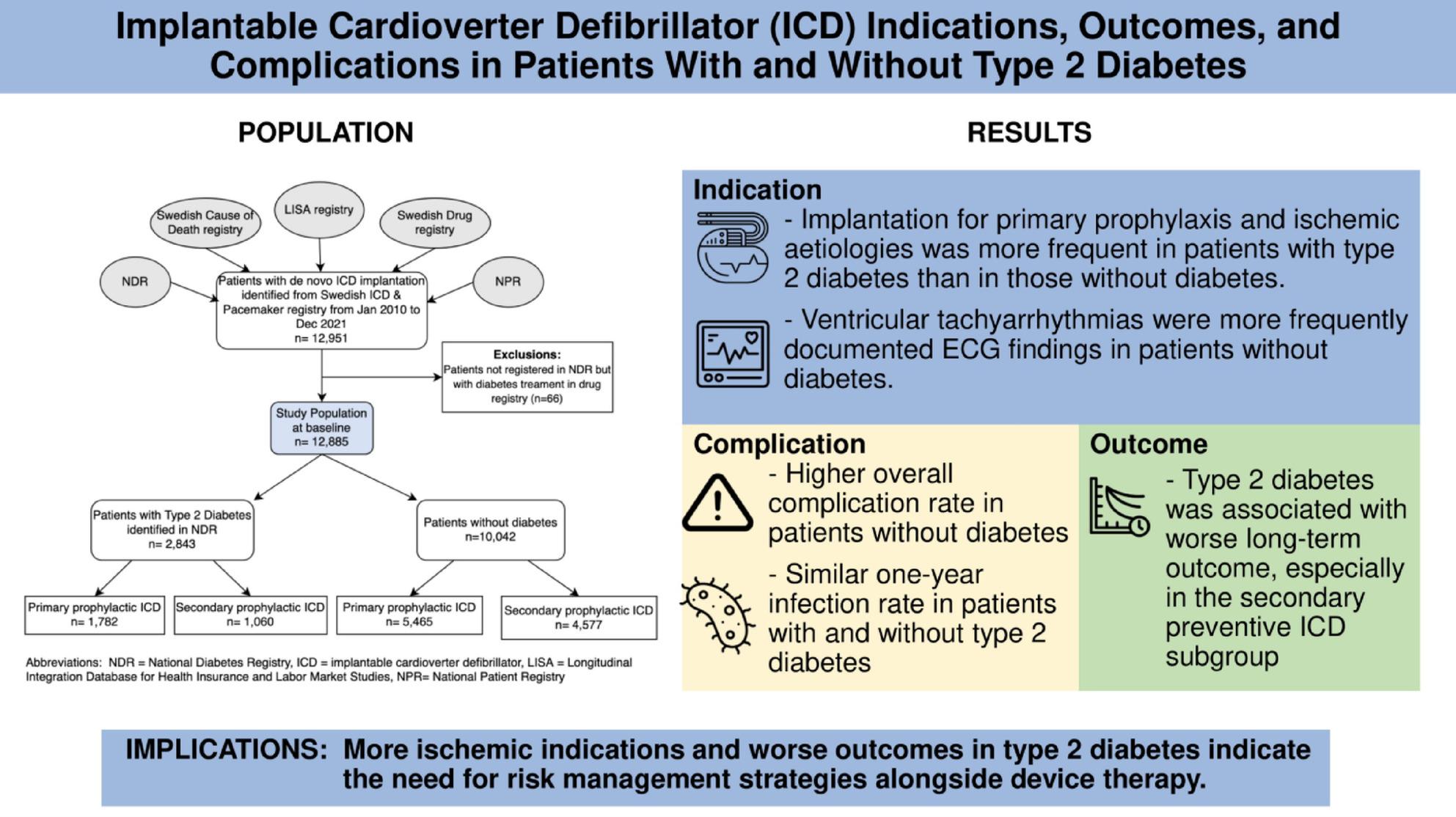

**Supplementary Information:**

The online version contains supplementary material available at 10.1186/s12933-025-03044-5.

## Research insights

**What is currently known about this topic?**

- Type 2 diabetes increases the need of implantable cardioverter defibrillators (ICD).
- Evidence is conflicting on whether type 2 diabetes increases ICD-related complication risk.
- ICDs improve outcomes in high-risk patients but data in diabetes are limited.

**What is the key research question?**

Do ICD indications, complications, and outcomes differ between patients with and without type 2 diabetes, and across ICD prevention types?

**What is new?**

- Type 2 diabetes patients more often received ICDs for primary and ischemic indications.
- One-year infection rates were similar in patients with and without type 2 diabetes.
- Worse prognosis in type 2 diabetes patients, especially in secondary prevention subgroups.

**How might this study influence clinical practice?**

Indicates the need for tailored risk management in type 2 diabetes patients beyond device therapy.

## Background

Patients with type 2 diabetes have an increased risk of tachyarrhythmias, leading to a higher need for implantation of a Cardioverter Defibrillator (ICD) [1]. The mechanisms behind this increased risk and whether the indications for ICD implantation or the underlying etiology differ between patients with type 2 diabetes and those without is unclear. Several factors may contribute, and one potential explanation is the presence of morphological changes in the heart, such as deposition of glycoproteins, interstitial edema, myocyte hypertrophy, and myocyte loss, which may arise from longstanding dysglycemia [2]. The higher prevalence of ischemic heart disease (IHD) among individuals with diabetes and a blunting of the autonomous nervous balance may also play a role [3]. Moreover, severe hypoglycemia, a dreaded complication associated with tight glycemic control, has been associated with an increased risk of life-threatening ventricular arrhythmias (both bradyarrhythmia and tachyarrhythmias) and sudden cardiac death (SCD) [4–7].

An ICD can be implanted for primary or secondary prevention of SCD [8]. Previous studies have shown a comparable all-cause mortality between ICDs implanted for primary versus secondary prevention, without specifying whether patients had diabetes or not [9–12]. The randomized trial DANISH (The Danish Study to Assess the Efficacy of ICDs in Patients With Nonischemic Systolic Heart Failure on Mortality) showed that patients with diabetes may derive less benefit from primary prophylactic ICD therapy compared to those without diabetes, in terms of reducing all-cause mortality [13]. However, data on the specific impact of ICDs in people with type 2 diabetes remains limited, including whether patients with diabetes may have different indications for the device compared to those without diabetes. Considering the high prevalence of IHD and heart failure (HF) among patients with diabetes, one might speculate that these conditions are frequent indications for ICD implantation among this patient group.

Given the clinical profile of this patient group, the risks and complications associated with ICD implantation remain a subject of debate. Diabetes was found to be an independent risk factor for device-related infection in a French case-control study of 2496 patients with follow-up of up to seven years, as well as in an American retrospective study of 2891 patients with six months of follow-up [14, 15]. In contrast, the DANISH trial reported that patients with diabetes had a similar risk of device infection (6.1% vs. 4.6%, *p* = 0.54) and other ICD-related complications compared to patients without diabetes [2].

In summary, the impact of diabetes on ICD indications, postoperative complications, and long-term outcomes remains unclear. The aim of this study was to investigate whether ICD indications differ between patients with and without type 2 diabetes, including in subgroups based on ICD prevention types. Another aim was to compare complications and prognosis after ICD implantation in these groups.

## Research design and methods

### Study cohort and data sources

This retrospective cohort study included patients aged ≥ 18 years registered in the Swedish ICD and Pacemaker Registry with a newly implanted ICD between January 2010 and December 2021. The entry point for the study was the date of ICD implantation, and patients were followed until December 2021 or until the time of death. The present study did not include patients with cardiac resynchronization therapy with defibrillators to ensure a clinically homogeneous cohort of ICD recipients and to enable more interpretable subgroup analyses by diabetes status and prevention type. After identifying the study population (*n* = 12,885) from the Swedish ICD and Pacemaker registry, specific information/variables from additional five registries were merged into a single dataset using personal identity numbers assigned to all individuals residing in Sweden. After merging, the data was pseudonymized, with each subject receiving a unique serial number to ensure confidentiality. Patients with records of diabetes medication use in the Swedish Drug registry, but without a corresponding diabetes diagnosis in the Swedish National Diabetes Registry (NDR), were excluded (*n* = 66).

The registries used in this study included six national health and population registries. The Swedish ICD and Pacemaker Registry holds data on 97% of all ICD implantations in Sweden, including implantation dates and indications. The Swedish NDR provides nationwide clinical data on adults with diabetes, including metabolic parameters, complications, and treatments, with a coverage of approximately 87%. The Longitudinal Integration Database for Health Insurance and Labor Market Studies (LISA), maintained by Statistics Sweden, includes annual socioeconomic data for all residents aged ≥ 16 years. The National Patient Register (NPR) captures data on inpatient care since 1964 and outpatient specialist visits since 2001, including diagnoses and procedures using International Classification of Diseases 10th revision (ICD-10) codes. The Swedish Prescribed Drug Register contains data on all prescribed medications dispensed at pharmacies nationwide. Finally, the Swedish Cause of Death Register provides complete mortality data, including both underlying and contributing causes of death based on ICD-10 codes.

### Definitions

Type 2 diabetes was defined based on a clinical diagnosis recorded in the NDR within 12 months prior to the entry date. The control group, referred to as No-DM patients, consisted of individuals not registered in the NDR, indicating the absence of any type of diabetes at the time of ICD implantation.

#### Baseline characteristics

Comorbidities at baseline, such as arrhythmias, HF, ventricular tachycardia/fibrillation (VT/VF), IHD and stroke, were identified from NPR using ICD-10 codes with diagnoses recorded at any point prior to study inclusion (specified in Table S1). Baseline medical treatments were defined as at least three dispensed prescriptions in the prescribed drug register within a 12-months period for each drug through Anatomical Therapeutic Chemical (ATC) code (Table S1). Socioeconomic variables were retrieved for all patients from LISA the year closest to the event year with available data, up to three years prior to the event to minimize missing data points and thereby maximize result reliability. Educational level was categorized as < 10 years, 10–12 years, or > 12 years. Marital status was categorized as single, divorced, married, or widowed. Country of birth was categorized as Sweden, Europe or outside of Europe. For individuals with type 2 diabetes, clinical characteristics were retrieved from the NDR using the measurement closest to the index date, taken within 365 days prior (definitions of the individual variables further specified in the Supplementary Material).

#### Indications and complications

Indications were registered at the time of implantation in the Swedish ICD and Pacemaker registry and categorized as symptoms, underlying etiology and ECG findings. Classifications of primary or secondary prevention for device implantation are defined based on a combination of symptoms and ECG-findings (see Supplementary Material). Complications within one year post-implantation were also retrieved from the Swedish ICD and Pacemaker Registry. 

#### Outcomes

The prognostic outcomes were all-cause mortality or MACE (major adverse cardiovascular events), defined as the first occurrence of non-fatal myocardial infarction (MI) (ICD-10 codes: I21), non-fatal stroke (I61, I63, I64), or CV death (I20-25, I61, I63, I64, I67.9). A stroke or MI was considered non-fatal if the patient survived for at least 28 days following diagnosis. Outcomes were identified using ICD-10 codes recorded in the NPR and the Swedish Cause of Death Registry, which is a reliable validated alternative to revised hospital discharge records and death certificates [16]. MACE and all-cause mortality were assessed based on time to event, expressed in years.

### Statistical analysis

The primary analysis of this study compared patients with type 2 diabetes with No-DM patients receiving an ICD (named as the main cohort below). Subsequently, differences were examined between patients with type 2 diabetes and No-DM patients receiving an ICD for primary or secondary prevention respectively. Finally, the study compared patients with type 2 diabetes receiving an ICD for primary versus secondary prevention, with a similar analysis conducted for No-DM patients.

Baseline characteristics from different registries were outlined for the patient groups described above. Continuous variables were presented as means and standard deviations (SD) and categorical data as numbers (n) and percentages (%). The p-values were assessed using t-tests for continuous and chi-square tests for categorical variables.

In the main cohort and in the subgroups with primary and secondary prevention, indications for ICD-implantation were presented as numbers (n) and percentages (%) in people with type 2 diabetes vs. No-DM. P-values were assessed using the chi-square test. Postoperative complications were presented as numbers (n) and percentages (%) and assessed using the chi-square test only in patients with type 2 diabetes vs. No-DM cohort due to low event rates.

The risk of MACE and all-cause mortality was assessed by Cox proportional hazard regression models and presented as hazard ratio (HR) with 95% confidence intervals (CI), both in the main cohort and the different subgroups. An interaction term between diabetes status and prevention type was included in the Cox regression models to formally test for interaction. Adjustments were performed in two models: Model 1: age, sex, marital status, educational level and country of birth (Sweden, Europe, outside Europe); Model 2: All variables in Model 1, together with IHD, HF, atrial fibrillation, peripheral artery disease, stroke and chronic obstructive pulmonary disease. The cumulative risk of MACE or all-cause mortality was illustrated using Kaplan–Meier curves and assessed with the log-rank test. Pairwise comparisons using the log-rank test were conducted based on diabetes status and prevention types. Absolute event rates (events per 100 person-years) were calculated for MACE and mortality by diabetes status and prevention type. Causes of death were categorized according to ICD-10 chapters using data from the national Cause of Death Register and summarized descriptively.

Sensitivity analyses using Cox regression model adjusted for calendar time were performed to account for evolving treatment patterns, and Fine–Gray model for MACE was applied to both the main comparison and subgroup analyses to account for competing risks. These adjustments did not significantly affect the results and are therefore not presented.

Due to a low proportion of missing data (< 1%), cases with missing covariates were excluded from the analyses without imputation. The study employed multiple hypothesis testing, where each hypothesis was analyzed separately and the existence of patterns in and the consistency of the results were considered in the analysis.

All analyses were performed using SAS 9.4. A two-sided p-value < 0.05 was considered statistically significant.

## Results

### Baseline characteristics

#### Main cohort

The study cohort consisted of 12,885 patients, 2,843 with type 2 diabetes and 10,042 No-DM patients at baseline, presented in Table 1. The mean age was higher in the type 2 diabetes group (67.9 ± 8.7 years) compared to No-DM patients (62.1 ± 14.3 years, *p* < 0.0001). A greater proportion of patients with type 2 diabetes were males (85.4% vs. 78.1%, *p* < 0.0001).

**Table 1:** Baseline characteristics at the time of ICD-implantation for type 2 diabetes and No-DM patients

	ICD (*n* = 12885)		*p*-value
	Type 2 diabetes *n* = 2843 (22.1%)	No DM *n* = 10,042 (77.9%)	
Patient characteristics			
Age at baseline (years)	67.9(8.7)	62.1(14.3)	< 0.0001
Male sex (%)	2428 (85.4)	7847 (78.1)	< 0.0001
Duration of Diabetes(years)	10.5 (8.7)	–	
BMI (kg/m^2^)	30.1 (5.2)	–	
Smoking n (%)	251 (14.0)	–	
HbA1c			
mmol/mol %	56.7 (14.9) 7.4 (3.5)	–	
eGFR (mL/min/1.73m^2^)	72.5 (25.1)	–	
Cholesterol (mmol/L)			
Total	4.1 (1.2)	–	
LDL	2.2 (0.9)	–	
HDL	1.1 (0.3)	–	
Triglycerides (mmol/L)	1.9 (1.4)	–	
Microalbuminuria n (%)	373 (25.4)	–	
Macroalbuminuria n (%)	150 (10.2)	–	
Systolic blood pressure (mmHg)	126.8 (17.8)	–	
Diastolic blood pressure (mmHg)	73.6 (10.1)	–	

	ICD (*n* = 12885)		*p*-value
	Type 2 diabetes *n* = 2843 (22.1%)	No DM *n* = 10,042 (77.9%)	
Medical history			
LBBB	4 (0.14)	18 (0.2)	0.660
Atrial flutter/fibrillation	615 (21.6)	1720 (17.1)	< 0.0001
Ventricular tachycardia/fibrillation	907 (31.9)	3859 (38.4)	< 0.0001
AV block			
I	6 (0.2)	19 (0.2)	0.815
II	28 (1.0)	76 (0.8)	0.230
III	51 (1.8)	192 (1.9)	0.683
Sick Sinus Syndrome	38 (1.3)	135 (1.3)	0.975
Ischemic Heart Disease	1663 (58.5)	3845 (38.3)	< 0.0001
Stroke	161 (5.7)	365 (3.6)	< 0.0001
Heart failure	1613 (56.7)	3894 (38.8)	< 0.0001
Peripheral artery disease	73 (2.5)	87 (0.87)	< 0.0001
Cardiac arrest	286 (10.1)	1337 (13.3)	< 0.0001

	ICD (*n* = 12885)		*p*-value
	Type 2 diabetes *n* = 2843 (22.1%)	No DM *n* = 10,042 (77.9%)	
Medical treatment			
ASA	1197 (42.1)	2728 (27.2)	< 0.0001
Other antiplatelet drug	214 (7.5)	399 (4.0)	< 0.0001
Anticoagulants	833 (29.3)	2177 (21.5)	< 0.0001
Beta-blocker	2086 (73.4)	5680 (56.6)	< 0.0001
RAS-acting agents	2207 (77.6)	5474 (54.5)	< 0.0001
Calcium antagonists	542 (19.1)	878 (8.7)	< 0.0001
Diuretics (thiazide and loop)	1134 (39.9)	1831 (18.2)	< 0.0001
MRA and other potassium sparing agents	821 (28.9)	1775 (17.7)	< 0.0001
Digoxin	166 (5.8)	325 (3.2)	< 0.0001
Antiarrhythmic drugs Class I and III	101 (3.6)	414 (4.1)	0.169
Ivabradin	10 (0.4)	17 (0.2)	0.058
Lipidlowering agents			
Statins	1881 (66.2)	3594 (35.8)	< 0.0001
Ezetimibe	151 (5.3)	307 (3.1)	< 0.0001
PCSK9I	2 (0.1)	11 (0.1)	0.567
Metformin	1177 (41.4)	–	
Sulfonylurea	227 (8.0)	–	
Combinations of oral glucose lowering agents	37 (1.3)	–	
Dipeptidylpeptidas-4-Inhibitor	186 (6.5)	–	
Insulin analog	876 (30.8)	–	
GLP-1 analog	203 (7.1)	–	
SGLT2-Inhibitor	170 (6.0)	–	

	ICD (*n* = 12885)		*p*-value
	Type 2 diabetes *n* = 2843 (22.1%)	No DM *n* = 10,042 (77.9%)	
Socio-economic status			
Marital status			
Married	1576 (55.4)	5459 (54.4)	0.311
Single	489 (17.2)	2259 (22.5)	< 0.0001
Divorced	551 (19.4)	1694 (16.9)	0.002
Widowed	222 (7.8)	604 (6.0)	0.001
Educational level			
<10 years	1019 (35.8)	2596 (25.9)	< 0.0001
10–12 years	1308 (46.0)	4479 (44.6)	0.184
>12 years	483 (17.0)	2845 (28.3)	< 0.0001
Country of birth			
Sweden	2289 (80.5)	8541 (85.1)	< 0.0001
Europe except Sweden	371 (13.1)	1028 (10.2)	< 0.0001
Outside Europe	183 (6.4)	473 (4.7)	0.0002

Categorical variables are presented as n (%) and continuous variables as mean (SD)

Variables including duration of diabetes, BMI, smoking status, HbA1c, eGFR, cholesterol, triglycerides, albuminuria, and systolic/diastolic blood pressure were retrieved from the National Diabetes Register and were therefore only available for patients with type 2 diabetes

ASA: Acetylsalicylic Acid, AV Block: Atrioventricular Block, eGFR: Estimated Glomerular Filtration Rate, GLP-1: Glucagon-Like Peptide-1, LBBB: Left Bundle Branch Block, MRA: Mineralocorticoid Receptor Antagonist, No-DM: No Diabetes Mellitus, PCSK9I: Proprotein Convertase Subtilisin/Kexin Type 9 Inhibitor, RAS: Renin-Angiotensin System, SGLT2: Sodium-Glucose Cotransporter-2

Based on data from the NPR, patients with type 2 diabetes had a significantly higher prevalence of cardiovascular diseases compared to No-DM patients, e.g. IHD (58.5% vs. 38.9%) and HF (56.7% vs. 39.4%). A history of atrial flutter/fibrillation (21.6% vs. 17.1%) was more frequent among patients with type 2 diabetes, while VT/VF (31.9% vs. 38.4%) and cardiac arrest (10.1% vs. 13.3%) were more common among No-DM patients (all *p* < 0.0001; Table 1). Pharmacological treatment was more prevalent among patients with type 2 diabetes, as regards antihypertensive medications, diuretics and anticoagulants, while the use of antiarrhythmic drugs did not differ.

#### Type 2 diabetes vs. No-DM in subgroups by prevention status

Similar baseline patterns as in the main cohort were observed in the subgroups receiving an ICD for primary (PP) or secondary (SP) prevention separately for patients with type 2 diabetes vs. without diabetes (Supplemental Table S2). For example, patients with type 2 diabetes were older than No-DM patients among both PP (66.9 vs. 62.1 years, *p* < 0.0001) and SP (69.6 and 62.0 years, *p* < 0.0001) ICD recipients. In the PP ICD subgroup, 61.4% of patients with type 2 diabetes had IHD compared to 42.7% of No-DM patients, while in the SP ICD group, the prevalence of IHD was 53.6% versus 32.9% (both *p* < 0.0001). Similarly, HF was more common in patients with type 2 diabetes, 74.0% vs. 56.2% in the PP ICD group and 27.8% vs. 17.9% in the SP ICD group (both *p* < 0.0001).

#### Primary vs. secondary prevention in subgroups by diabetes status

Supplemental Table S2 also presents p-values in between prevention types in patients with and without type 2 diabetes respectively. In general, PP ICD recipients had more cardiovascular comorbidities, and the use of nearly all described medications was more prevalent compared to SP patients regardless of diabetes status.

### Indications

#### Main cohort

Indications (ECG-findings, etiology and symptoms) for ICD recipients, registered at the time of ICD implantation in the Swedish PM and ICD registry, are presented in Table 2. A higher proportion of PP ICDs were implanted in patients with type 2 diabetes compared to No-DM patients (62.7% vs. 54.5%, *p* < 0.0001).

**Table 2:** Comparison of indication for de-novo ICD implantation between type 2 diabetes and No-DM patients

	Type 2 diabetes *n* = 2843 (22.1%)	No-DM *n* = 10,042 (77.9%)	*p*-value
*Prevention Type* *s*			
Primary	1782 (62.7)	5512(54.5)	< 0.0001
Secondary	1060 (37.3)	4599 (45.5)	< 0.0001

	Type 2 diabetes *n* = 2843 (22.1%)	No-DM *n* = 10,042 (77.9%)	*p*-value
*Symptoms*			
Breathlessness/tiredness	218 (7.7)	664 (6.6)	0.040
Asymptomatic/Primary prophylaxis	964 (52.1)	2948 (49.9)	< 0.0001
Heart failure symptoms	417 (14.7)	1043 (10.3)	< 0.0001
Palpitations	179 (6.3)	814 (8.1)	0.002
Syncope	437 (15.4)	1932 (19.1)	< 0.0001
Aborted sudden death	628 (22.1)	2686 (26.6)	< 0.0001

	Type 2 diabetes *n* = 2843 (22.1%)	No-DM *n* = 10,042 (77.9%)	*p*-value
*Etiolog* *y*			
ARVC	6 (0.2)	265 (2.6)	< 0.0001
Amyloidosis	5 (0.2)	38 (0.4)	0.098
Other structural heart disease	89 (3.1)	584 (5.8)	< 0.0001
Idiopathic	202 (7.1)	1334 (13.3)	< 0.0001
Ischemic heart disease	1357 (47.7)	3277 (32.6)	< 0.0001
Dilated cardiomyopathy	435 (15.3)	1754 (17.5)	0.007
Hypertrophic cardiomyopathy	71 (2.5)	616 (6.13)	< 0.0001
Ischemic cardiomyopathy	462 (16.3)	999 (10.0)	< 0.0001
Valvular heart disease	17 (0.6)	119 (1.3)	0.007
Congenital heart disease	10 (0.4)	139 (1.4)	< 0.0001
Long QT syndrome	21 (0.7)	238 (2.4)	< 0.0001
Myocarditis	11 (0.4)	160 (1.6)	< 0.0001
Post-infarction	14 (0.5)	366 (3.6)	0.0003
Sarcoidosis	9 (0.3)	138 (1.4)	< 0.0001
Other^*^	2 (0.1)	16 (0.2)	0.395

	Type 2 diabetes *n* = 2843 (22.1%)	No-DM *n* = 10,042 (77.9%)	*p*-value
*EC* *G*			
VF	450 (15.8)	2054 (20.5)	< 0.0001
VT	598 (21.0)	2604 (25.9)	< 0.0001
VT + VF	228 (8.0)	852 (8.5)	0.430
NSVT	121 (4.3)	503 (5.0)	0.099
Primary prophylaxis	1445 (50.8)	4027 (40.1)	< 0.0001

Indications are categorized as etiology, symptoms and ECG-findings from Swedish ICD and pacemaker registry. Presented as n (%)

ARVC: Arrhythmogenic Right Ventricular Cardiomyopathy, No-DM: No Diabetes Mellitus, NSVT: Non-Sustained Ventricular Tachycardia, VT/VF: Ventricular Tachycardia/Ventricular Fibrillation

*Other included Enodcarditis, High proportion of right ventricular pacing, Cytostatic-induced cardiomyopathy, Post TAVI (Transcatheter Aortic Valve Implantation) and conduction system fibrosis

Regarding ECG findings, No-DM patients had a higher reported prevalence of VF (15.8% vs. 20.5%, *p* < 0.0001) and VT (21.0% vs. 25.9%, *p* < 0.0001), compared to patients with type 2 diabetes.

In terms of underlying etiology for ICD-implantation, IHD was the most common reason for ICD implantation regardless of the presence of type 2 diabetes or not. IHD was, however, reported more often as an indication among those with type 2 diabetes (47.7% vs. 32.6%, *p* < 0.0001). Non-vascular conditions as indications were more prevalent among No-DM patients compared to those with type 2 diabetes, e.g. ARVC (arrhythmogenic right ventricular cardiomyopathy; 0.2% vs. 2.6%), other structural cardiac diseases (3.1% vs. 5.8%), long QT syndrome (0.7% vs. 2.4%), sarcoidosis (0.3% vs. 1.4%), and dilated cardiomyopathy (15.3% vs. 17.5%).

The most common symptoms registered in both type 2 diabetes and No-DM patients were aborted sudden death, followed by HF-symptoms and syncope. HF-symptoms (14.7% vs. 10.3%, *p* < 0.0001) and breathlessness/fatigue (7.7% vs. 6.6%, *p* = 0.040) were more prevalent among type 2 diabetes patients compared to No-DM.

#### Type 2 diabetes vs. No-DM in subgroups by prevention status

In the PP subgroup, IHD (type 2 diabetes: 44.2%; No-DM 31.4%, *p* < 0.0001) was, as in the main patient cohort, the dominant etiology in both type 2 diabetes and No-DM patients, followed by dilated cardiomyopathy (type 2 diabetes: 19.8%; No-DM 23.8%, *p* = 0.001) and ischemic cardiomyopathy (type 2 diabetes: 19.3%; No-DM 13.2%, *p* < 0.0001) (Supplemental Table S3). Like the main cohort, structural cardiomyopathies were more common as an indication in No-DM patients. In terms of underlying symptoms, HF-symptoms (type 2 diabetes 14.7% vs. No-DM 10.3%, *p* < 0.0001) and palpitations (type 2 diabetes 6.3% vs. No-DM 8.1%, *p* = 0.002) were the most common. ECG findings showed a similar pattern as for the main group.

In the SP subgroup, IHD (type 2 diabetes: 53.7% vs. No-DM 34.1%, *p* < 0.0001) and ischemic cardiomyopathy (type 2 diabetes: 11.2% vs. No-DM 6.0%, *p* < 0.0001) were the most common causes both in type 2 diabetes and No-DM patients (Supplemental Table S3). However, a notable proportion of patients receiving secondary prophylactic ICDs had an idiopathic etiology, particularly in the No-DM group (21.4%). No significant differences in symptoms and ECG-findings were observed based on diabetes status.

### Complications

The overall complication rate was higher among No-DM patients (5.9% vs. 7.1%, *p* = 0.035) during the one-year follow-up (Table 3). The prevalence of post-operative infections did not differ between type 2 diabetes and No-DM patients. However, local bleeding was more common in those with type 2 diabetes (0.8% vs. 0.3%, *p* = 0.001), while electrical dysfunction was slightly more prevalent among No-DM patients (1.1% vs. 1.7%, *p* = 0.035). No further analysis was performed in subgroups due to the low complication rates.

**Table 3:** Comparison of one-year complication after de-novo ICD-implantation between type 2 diabetes and No-DM patients

	Type 2 diabetes *n* = 2843 (21.9%)	No-DM *n* = 10,042 (78.1%)	*p*-value
Aborted procedure	15 (0.6)	39 (0.4)	0.603
Local bleeding	22 (0.8)	33 (0.3)	0.001
Electrical dysfunction (e.g. lead failure, pacing abnormality)	32 (1.1)	170 (1.7)	0.035
Lead dislocation	39 (1.4)	172 (1.7)	0.220
Infection	32 (1.1)	135 (1.3)	0.381
Perforation/Tamponade	10 (0.4)	45 (0.4)	0.587
Pneumothorax	6 (0.2)	33 (0.3)	0.321
Venous embolism/thrombosis	0 (0.0)	10 (0.1)	0.093
Other	13 (0.5))	78 (0.8)	0.093
**Total**	**169 (5.9)**	**715 (7.1)**	**0.035**

Presented as n (%)

### Prognosis after ICD implantation

#### Main cohort

**Fig. 1:**
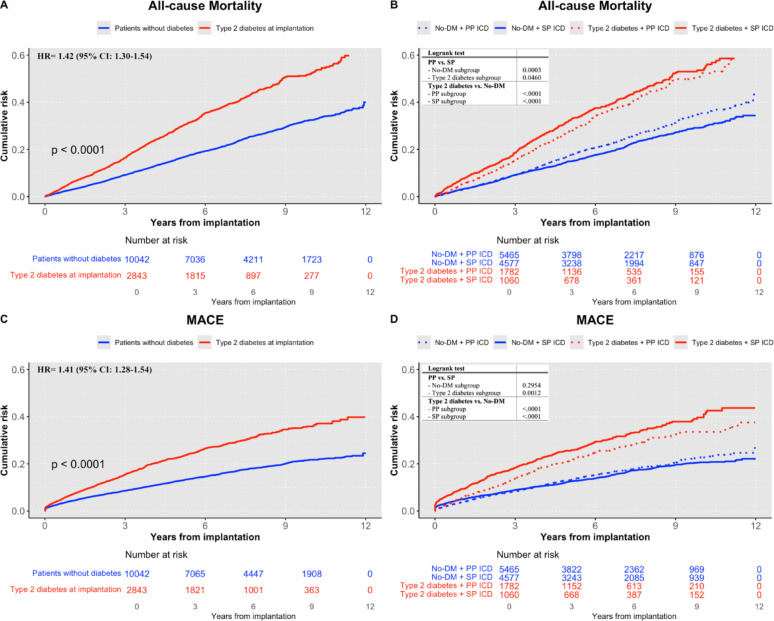
Cumulative risk of MACE and all-cause mortality after de-novo ICD implantation, stratified by type 2 diabetes status and prevention groups. Cumulative risk of all-cause mortality after ICD implantation in **a** patients with and without Type 2 Diabetes Mellitus and **b** stratified by type 2 diabetes status and prevention type. Cumulative risk of Major adverse cardiovascular event (MACE) after ICD implantation in **c** patients with and without type 2 diabetes and **d** stratified by type 2 diabetes status and prevention type. Numbers below the figure represent individuals at risk. Hazard ratio (HR) adjusted for sex, age, marital status, education, country of birth and previous ischemic heart disease and heart failure. Abbreviations: No-DM: patients without diabetes. PP: primary prevention, SP: secondary prevention

The mean follow-up time for the main cohort was 5.2 ± 3.3 years (type 2 diabetes: 4.6 ± 3.0; No-DM: 5.3 ± 3.3). Patients with type 2 diabetes had a higher cumulative risk of all-cause mortality and MACE (Fig. 1A + C) after ICD implantation, with a similar distribution of causes of death between groups (Supplemental Table S4). This difference was apparent soon after implantation and became more pronounced over time. Cox regression analysis confirmed that patients with type 2 diabetes had a significantly higher risk of MACE (unadjusted HR: 1.87 [95% CI: 1.71–2.05]) and all-cause mortality (HR: 1.95 [1.81–2.11]), persisting after adjustments for potential confounders in the two models (Supplemental Table S5).

#### Type 2 diabetes vs. No-DM in subgroups by prevention status

In both prevention subgroups, patients with type 2 diabetes had a significantly increased risk of MACE and all-cause mortality (Fig. 1B + D), which remained significant after adjustment in model 2 for demographic and clinically relevant comorbidities (Supplemental Table S6). In the PP group, the adjusted HR was 1.31 [1.15–1.48] for MACE and 1.34 [1.21–1.49] for all-cause mortality. Notably, the risk was even higher in the SP group, with an adjusted HR of 1.54 [1.34–1.78] for MACE and 1.55 [1.37–1.76] for all-cause mortality compared to No-DM patients. Formal interaction between diabetes status and prevention type was statistically significant for all-cause mortality (*p* = 0.0003) and MACE (*p* = 0.005).

#### Primary vs. secondary prevention in subgroups by diabetes status

Within the type 2 diabetes subgroup, patients with ICDs for PP had a lower risk for both MACE (*p* = 0.001) and all-cause mortality (*p* = 0.046) compared to those with SP (Fig. 1B + D). This was consistent for the individual MACE components (cardiovascular death, non-fatal MI and stroke). In Cox regression analysis (Supplemental Table S7), adjusted according to model 2, PP patients with type 2 diabetes had a lower risk for both MACE (HR: 0.75 [0.63–0.89]) and all-cause mortality (HR: 0.79 [0.68–0.91]).

In contrast, within No-DM patients, MACE risk was similar for PP and SP as outlined in Fig. 1B and Supplemental Table S7. No-DM patients who received ICDs for PP had a higher risk for all-cause mortality compared to SP (HR: 1.18 [1.08–1.29] in the unadjusted analyses but this was however blunted after adjustments in model 2 (Supplemental Table S7). Corresponding absolute event rates for MACE and all-cause mortality, stratified by diabetes status and prevention types, are provided in Supplementary Table S8.

## Conclusion

The main findings of this study are that the indications for ICD implantation and the subsequent prognosis in patients with type 2 diabetes differ from No-DM patients. While PP ICD-implantations were more common in patients with type 2 diabetes, presumably due to a higher prevalence of IHD and HF, SP was more frequent in No-DM patients and more often due to non-ischemic or genetic conditions (i.e. ARVC, long QT syndrome, other structural cardiac diseases). The latter conditions might debut abruptly, leading to life-threatening arrhythmia that require ICD implantation for SP. Patients with type 2 diabetes had a higher adjusted risk of all-cause mortality and MACE, underlining the dismal prognostic implications of type 2 diabetes. Finally, the risk of infections during a one-year follow-up was comparable between the groups.

IHD was the most common etiology for ICD implantation in both type 2 diabetes and No-DM patients, although it was more prevalent in the type 2 diabetes group. This is in accordance with a higher risk for IHD in patients with type 2 diabetes. One quarter of Swedish patients hospitalized with acute myocardial infarction have known diabetes and about one third of those with previously undetected type 2 diabetes have it if properly screened, which contributes to their increased prevalence of IHD which contributes to the need for ICD implantation [17, 18]. Preventing IHD should reasonably reduce this need, underscoring the importance of effective cardiovascular risk management not the least in this high-risk population [19]. Ventricular tachyarrhythmias were less frequently recorded as an ECG-indication for type 2 diabetes patients, despite their reported higher arrhythmic risk [4, 5]. This suggests that their greater burden of comorbidities contributed to PP ICD implantation prior to an outburst of ventricular tachyarrhythmia.

The poorer outcomes observed in patients with type 2 diabetes persisted after adjusting for demographic variables and clinically relevant comorbidities. This suggests that other diabetes-related mechanisms, such as chronic inflammation, metabolic changes, and vascular dysfunction, contribute to the dismal outcome. Another perspective to consider is whether differences in the etiology of ICD implantation between patients with and without type 2 diabetes might influence prognosis. Previous studies have indicated increased risk of all-cause mortality among patients receiving ICDs for ischemic cardiomyopathies [20, 21]. Patients with ischemic indications had a similar risk of life-threatening ventricular arrhythmia, but an increased risk of non-sudden cardiac death compared to patients with a non-ischemic background to the ICD treatment [21].

In the type 2 diabetes subgroup, patients with SP had significantly worse outcomes, with higher rates of all-cause mortality, compared to those receiving ICDs for PP. This is in similarity with a study by Liu et al. reporting higher all-cause mortality in SP patients regardless type 2 diabetes status [22]. A potential reason may be that individuals with type 2 diabetes subjected to SCD often had a non-shockable rhythm (e.g. pulseless electrical activity), reasonably due to higher comorbidity burden [23, 24]. Recent study also underscores the need for more refined SCD risk stratification in diabetes, including electrocardiographic markers such as the QRS–T angle, which is associated with higher SCD-risk [25].

Patients receiving ICDs for PP often face a higher risk of non-arrhythmic deaths reducing the efficacy of ICDs [12, 26, 27]. This gains support from the present finding that the differing mortality risk between PP and SP subgroups in No-DM patients diminished following adjustment for clinically relevant comorbidities, including HF and IHD. Thus, it may be the higher prevalence of comorbidities, rather than the prevention type, that drives the increased mortality in patients with PP ICDs. Regarding the risk of MACE, there was no significant difference between patients receiving ICD for PP and SP in the No-DM group. However, in the type 2 diabetes group the risk for MACE was higher if the indication was SP. This may be due to the increased risk of non-cardiac events among PP patients [26, 27]. Given the high mortality in this cohort, sensitivity analysis using Fine and Gray models confirmed that accounting for death as a competing event did not materially change the interpretation of our findings, supporting the robustness of the main results.

The infection rate in this study was comparable with previous studies on ICD, around 1–2% during the first year after implantation [14]. Of note is that the overall risk of postoperative complications was numerically higher in No-DM patients and the infection rate was similar in type 2 diabetes and No-DM patients. This is in contrast with a previous systematic review, which primarily included case-control studies and identified diabetes as a risk factor for increased postoperative infections after cardiac device implantation (OR 95% CI 2.08 [1.62–2.67] [28]. However, a later retrospective cohort study by El-Chami et al. reported no significant increase in the risk of infection among patients with uncomplicated diabetes after ICD implantation. They did observe a slightly higher odds ratio for mid- (91–365 days) and late (366–720 days) device infections among patients with complicated diabetes [29]. Notably, our finding is supported by a large-scale study based on Medicare patient data that suggests diabetes, by itself, may not be an independent risk factor for ICD-related infections [30]. Instead, other factors such as renal failure, chronic pulmonary and cerebrovascular disease may play a more prominent role. In the present study, local bleeding was more common in patients with type 2 diabetes, potentially due to vascular changes and impaired wound healing associated with type 2 diabetes [31, 32] or simply explained by the higher use of anticoagulants in this patient group.

One of the strengths of the present study is the large sample size reflecting real-world clinical practice and minimizing selection bias. This allowed for the creation of stratified subgroup comparisons, which have rarely been conducted in previous studies in this area. The use of high-quality nationwide registries further enhances the reliability of our findings [33]. A recently published review of the NPR has shown diagnostic accuracy with a median positive predictive value of 84% for evaluated diagnoses [34]. Another strength is the focus on patients with type 2 diabetes. Most previous studies did not differentiate between diabetes types. Diabetes is a heterogeneous condition, and type 1 diabetes, for instance, is associated with a higher rate of glycemic variability and severe hypoglycemic episodes, both of which negatively impact mortality risk [35, 36]. Additionally, glycemic variability appears to be linked to plaque vulnerability and subclinical coronary atherosclerosis [36]. A more homogeneous group of type 2 diabetes patients allows for a more accurate interpretation of the results, minimizing confounding factors related to different diabetes subtypes.

Certain limitations should be acknowledged, including the inability to fully account for potential confounders due to the observational and registry-based design of the study. Several device-related variables were not available in the extracted dataset, such as single- vs. dual-chamber configuration, HF-related variables (ejection fraction) and ICD therapy data (appropriate/inappropriate shocks, QRS-duration). These factors limit our ability to explore some of the device-specific mechanisms that may contribute to complications or outcomes. Moreover, under-reporting of minor complications in registry data is possible and may vary across patient subgroups, which could lead to an underestimation of complication rates and potentially impair comparisons between subgroups. There are gaps and inconsistencies between the registries used. For example, some patients listed as receiving diabetes medications in the drug registry were not classified as having diabetes according to the NDR. To maintain clarity in group categorization, such patients were excluded. Furthermore, the present study was conducted during a period of rapid therapeutic evolution. Although calendar time was adjusted for in sensitivity analyses, residual confounding cannot be fully excluded.

In conclusion, in patients receiving de novo ICDs, PP implantation was more frequent among those with type 2 diabetes than in No-DM patients. The most likely reason is their higher prevalence of comorbidities such as IHD and HF. Ventricular tachyarrhythmias were less frequently recorded as ECG indications for type 2 diabetes patients, despite their previously reported elevated arrhythmic risk. No signal of increased infection risk associated with type 2 diabetes was observed in this real-world cohort, but type 2 diabetes was associated with worse long-term outcomes, underscoring the need for comprehensive risk management.

## Supplementary Information

Below is the link to the electronic supplementary material.

Supplementary Material 1

## Data Availability

The datasets generated during and/or analyzed in the current study are available from the corresponding author upon reasonable request.

## Abbreviations

ARVC: Arrhythmogenic Right Ventricular Cardiomyopathy
ATC: Anatomical Therapeutic Chemical
AV: Atrio-ventricular
CV-deaths: Cardiovascular deaths
CI: Confidence interval
eGFR: Estimated glomerulus filtration rate
GLP-1: Glucagon-like peptide 1
HbA1c: Glycated hemoglobin A1C
HF: Heart failure
HR: Hazard ratio
ICD: Implantable cardioverter defibrillator
ICD-10: International classification of diseases 10th revision
IHD: Ischemic heart disease
LISA: Longitudinal integration database for health insurance and labour market studies
MACE: Major Adverse Cardiovascular Event
MI: Myocardial infarction
NDR: National Diabetes Registry
No-DM: Patients without diabetes
NPR: National Patient Registry
PP: Primary prevention
SCD: Sudden cardiac death
SD: Standard deviation
SGLT-2: Sodium glucose linked co-transporter 2
SP: Secondary prevention
VF: Ventricular fibrillation
VT: Ventricular tachycardia
