# Supplementary material for: Indications, prognosis, and complications of de Novo implantable cardioverter defibrillators in patients with and without type 2 diabetes: a nationwide registry-based cohort study
Source: Cardiovasc Diabetol. 2025 Dec 31;25:27. doi: 10.1186/s12933-025-03044-5 (PMC12849419; doi:10.1186/s12933-025-03044-5)
Supplement: Supplementary file 1 — Supplementary Material 1 [file 12933_2025_3044_MOESM1_ESM.docx]

**Indications, Prognosis, and Complications of De Novo Implantable Cardioverter Defibrillators in Patients with and without Type 2 Diabetes: A Nationwide Registry-based Cohort Study**

**Supplementary appendix**

Tables of contents:

[Table S1 – ICD 10 Codes and ATC-codes used. 3](#_Toc215917076)

[Table S2- Baseline characteristics at the time of ICD-implantation for type 2 diabetes vs No-DM patients in subgroups receiving ICD for primary and secondary prevention. 4](#_Toc215917077)

[Table S3- Comparison of indication for de-novo ICD implantation between type 2 diabetes vs. No-DM patients in subgroups receiving ICD for primary and secondary prevention 6](#_Toc215917078)

[Table S4- Cause-of-death categories based on ICD-10 codes 7](#_Toc215917079)

[Table S6- Risk of MACE and all-cause mortality in type 2 diabetes vs. No-DM patients (reference) in subgroups receiving ICD for primary and secondary prevention 9](#_Toc215917080)

[Table S7- Risk of MACE and all-cause mortality in patients with primary vs. secondary (reference) prophylactic ICD based on diabetes status 10](#_Toc215917081)

[Table S8- Absolute event rates (events per 100 person-years) for MACE and mortality by type 2 diabetes status and prevention type 11](#_Toc215917082)

[Definitions of variables 12](#_Toc215917083)

[Classification of prevention types from Swedish ICD and Pacemaker Registry 13](#_Toc215917084)

# **Table S1 – ICD 10 Codes and ATC-codes used.**

|  | **ICD-10 and ATC codes** | **Comments** |
| --- | --- | --- |
| **Comorbidities (ICD)** |  | From the National Patient Registry |
| Ischaemic heart disease | (I20–I25) |  |
| AV-block I | I44.0 |  |
| AV-block II | I44.1 |  |
| AV-block III | I44.2 |  |
| LBBB | I44.6 |  |
| Cardiac arrest | I46 |  |
| Ventricular tachycardia | I47.2 |  |
| Atrial fibrillation/atrial flutter | I48 |  |
| Ventricular fibrillation | I49.0 |  |
| Sick sinus syndrome | I49.5 |  |
| Heart failure | I50 |  |
| Stroke | I61, I63, I64 |  |
| Peripheral artery disease | I73.9 |  |
| **Medication (ATC)** |  | From the National Drug Registry. Medications at baseline were defined as at least three filled prescriptions within 12 months prior implantation |
| Aspirin | B01AC04 |  |
| Other antiplatelet drug | B01AC06 (Clopidogrel) B02AC22(Prasugrel) B02AC24 (Ticagrelor) |  |
| Anticoagulants | B01AA (vitamin K-antagonist) B01AE (Pradaxa) B01AF (Direct factor Xa inhibitors) |  |
| Beta-blocker | C07A |  |
| RAS-acting agents | C09 |  |
| Calcium antagonists | C08 |  |
| Thiazide diuretics  Loop diuretics | C03A C03C |  |
| MRA and other potassium sparing agents | C03D |  |
| Digoxin | C01AA05 |  |
| Antiarrhythmic drugs Class I and III | C01B |  |
| Ivabrdin | C01EB17 |  |
| Lipidlowering agents - Statins - Ezetimib - PCSK9I | C10AA C10AX09 C10AX13, C10AX14, C10AX16 |  |
| Metformin | A10BA02 |  |
| Sulfonylurea | A10BB |  |
| Combinations of oral diabetes medications | A10BD |  |
| Dipeptidylpeptidas-4-Inhibitor | A10BH |  |
| Insulin analog | A10A |  |
| GLP-1 analog | A10BJ |  |
| SGLT2-Inhibitor | A10BK |  |

Abbreviations: ATC= Anatomical Therapeutic Chemical; ICD= implantable cardioverter defibrillator; LBBB = left bundle branch block; MRA: Mineralocorticoid Receptor Antagonist; RAS: Renin-Angiotensin System; PCSK9I: Proprotein Convertase Subtilisin/Kexin Type 9 Inhibitor; GLP-1: Glucagon-Like Peptide-1; SGLT2: Sodium-Glucose Cotransporter-2

**Table S2- Baseline characteristics at the time of ICD-implantation for type 2 diabetes vs No-DM patients in subgroups receiving ICD for primary and secondary prevention.**

|  | **Primary prevention (n= 7247)** | | **p-value** | **Secondary prevention (n= 5637)** | | **p-value** | **Type 2 diabetes** | **No DM** |
| --- | --- | --- | --- | --- | --- | --- | --- | --- |
|  | **Type 2 diabetes**  **n= 1782 (24.6%)** | **No DM**  **n= 5465 (75.4%)** |  | **Type 2 diabetes**  **n= 1060 (18.8%)** | **No DM**  **n= 4577 (81.2%)** |  | Primary vs. Secondary prevention | Primary vs. Secondary prevention |
| **Patient characteristics** | | | | | | | | |
| Age at baseline (years) | 66.9 (8.7) | 62.1 (13.6) | <0.0001 | 69.6 (8.5) | 62.0 (15.1) | <0.0001 | <0.0001 | 0.670 |
| Male sex (%) | 1519 (85.2) | 4300 (78.7) | <0.0001 | 908 (85.7) | 3547 (77.5) | <0.0001 | 0.760 | 0.152 |
| Duration of Diabetes(years) | 10.4 (8.5) | - |  | 10.7 (9.0) | - |  | 0.326 |  |
| BMI (kg/m2) | 30.1 (5.2) | - |  | 30.0 (5.1) | - |  | 0.542 |  |
| Smoking n (%) | 165 (14.3) | - |  | 86 (13.6) | - |  | 0.664 |  |
| HbA1c |  |  |  |  |  |  | 0.067 |  |
| - mmol/mol | 57.1 (14.9) | - |  | 55.8 (14.7) | - |  |  |  |
| - % | 7.4 (3.5) | - |  | 7.3 (3.5) | - |  |  |  |
| eGFR (mL/min/1.73m^2^) | 72.3 (24.7) | - |  | 72.7 (26.0) | - |  | 0.704 |  |
| Cholesterol (mmol/L) |  |  |  |  |  |  |  |  |
| -Total | 4.0 (1.2) | - |  | 4.2 (1.2) | - |  | 0.0006 |  |
| - LDL | 2.2 (0.9) | - |  | 2.4 (1.0) | - |  | 0.0004 |  |
| - HDL | 1.1 (0.3) | - |  | 1.1 (1.3) | - |  | 0.0003 |  |
| Triglycerides (mmol/L) | 2.0 (1.4) | - |  | 1.9 (1.3) | - |  | 0.278 |  |
| Microalbuminuria  n (%) | 247 (26.5) | - |  | 126 (23.6) | - |  | 0.569 |  |
| Macroalbuminuria  n (%) | 93 (10.0) | - |  | 57 (10.7) | - |  | 0.569 |  |
| Systolic blood pressure  (mmHg) | 124.1 (17.1) | - |  | 131.7 (17.9) | - |  | <0.0001 |  |
| Diastolic blood pressure (mmHg) | 73.1 (10.0) | - |  | 74.6 (10.3) | - |  | 0.002 |  |
| **Medical history** | | | | | | | | |
| LBBB | 4 (0.2) | 8 (0.1) | 0.481 | 0 (0.0) | 10 (0.2) | 0.1277 | 0.123 | 0.395 |
| Atrial flutter/fibrillation | 415 (23.3) | 1033 (18.9) | <0.0001 | 200 (18.9) | 687 (15.0) | 0.002 | 0.006 | <0.0001 |
| VT/VF | 316 (17.7) | 1248 (22.8) | <0.0001 | 591 (55.7) | 2611 (57.1) | 0.049 | <0.0001 | <0.0001 |
| AV block |  |  |  |  |  |  |  |  |
| I | 5 (0.3) | 14 (0.3) | 0.861 | 1 (0.1) | 5 (0.1) | 0.893 | 0.296 | 0.092 |
| II | 17 (1.0) | 47 (0.9) | 0.713 | 11 (1.0) | 29 (0.6) | 0.158 | 0.827 | 0.192 |
| III | 27 (1.5) | 120 (2.2) | 0.077 | 24 (2.3) | 72 (1.6) | 0.117 | 0.146 | 0.023 |
| Sick Sinus Syndrome | 22 (1.2) | 69 (1.3) | 0.927 | 16 (1.5) | 66 (1.4) | 0.869 | 0.537 | 0.437 |
| Ischemic Heart Disease | 1094 (61.4) | 2335 (42.7) | <0.0001 | 569 (53.6) | 1510 (32.9) | <0.0001 | <0.0001 | <0.0001 |
| Stroke | 101 (5.7) | 210 (3.8) | 0.0010 | 60 (5.7) | 155 (3.4) | 0.0005 | 0.993 | 0.224 |
| Heart failure | 1319 (74.0) | 3072 (56.2) | <0.0001 | 294 (27.8) | 822 (17.9) | <0.0001 | <0.0001 | <0.0001 |
| Peripheral artery disease | 45 (2.5) | 58 (1.1) | <0.0001 | 28 (2.6) | 29 (0.6) | <0.0001 | 0.850 | 0.021 |
| Cardiac arrest | 23 (1.3) | 75 (1.4) | 0.795 | 263 (24.8) | 1262 (27.6) | 0.068 | <0.0001 | <0.0001 |
| **Medical treatment** | | | | | | | | |
| ASA | 763 (42.8) | 1652 (30.2) | <0.0001 | 434 (40.9) | 1076 (23.5) | <0.0001 | 0.328 | <0.0001 |
| Other antiplatelet drug | 150 (8.4) | 288 (5.3) | <0.0001 | 64 (6.0) | 111 (2.4) | <0.0001 | 0.020 | <0.0001 |
| Anticoagulants | 591 (33.1) | 1420 (26.0) | <0.0001 | 242 (22.8) | 734 (16.0) | <0.0001 | <0.0001 | <0.0001 |
| Beta-blocker | 1426 (80.0) | 3617 (66.1) | <0.0001 | 659 (62.1) | 2063 (45.0) | <0.0001 | <0.0001 | <0.0001 |
| RAS-acting agents | 1481 (83.1) | 3547 (64.9) | <0.0001 | 725 (68.3) | 1927 (42.1) | <0.0001 | <0.0001 | <0.0001 |
| Calcium antagonists | 261 (14.7) | 365 (6.7) | <0.0001 | 281 (26.5) | 513 (11.2) | <0.0001 | <0.0001 | <0.0001 |
| Diuretics (thiazide and loop) | 821 (46.1) | 1242 (22.7) | <0.0001 | 312 (29.4) | 589 (12.9) | <0.0001 | <0.0001 | <0.0001 |
| MRA and other potassium sparing agents | 678 (38.1) | 1391 (25.5) | <0.0001 | 143 (13.5) | 384 (8.4) | <0.0001 | <0.0001 | <0.0001 |
| Digoxin | 111 (6.2) | 213 (3.9) | <0.0001 | 55 (5.2) | 112 (2.4) | <0.0001 | 0.253 | <0.0001 |
| Antiarrhythmic drugs Class I and III | 62 (3.5) | 220 (4.0) | 0.300 | 39 (3.7) | 194 (4.2) | 0.410 | 0.781 | 0.593 |
| Ivabradin | 9 (0.5) | 13 (0.2) | 0.075 | 1 (0.1) | 3 (0.1) | 0.566 | 0.102 | 0.042 |
| Lipidlowering agents |  |  |  |  |  |  |  |  |
| Statins | 1272 (71.4) | 2237 (40.9) | <0.0001 | 608 (57.3) | 1357 (29.6) | <0.0001 | <0.0001 | <0.0001 |
| Ezetimibe | 111 (6.2) | 217 (4.0) | <0.0001 | 40 (3.8) | 90 (2.0) | 0.0004 | 0.005 | <0.0001 |
| PCSK9I | 2 (0.1) | 9 (0.2) | 0.621 | 0 (0.0) | 2 (0.0) | 0.496 | 0.532 | 0.068 |
| Metformin | 763 (42.8) | - |  | 413 (39.0) | - |  | 0.044 |  |
| Sulfonylurea | 141 (7.9) | - |  | 86 (8.1) | - |  | 0.849 |  |
| Combinations of oral glucose lowering agents | 24 (1.4) | - |  | 13 (1.2) | - |  | 0.784 |  |
| Dipeptidylpeptidas-4-Inhibitor | 128 (7.2) | - |  | 57 (5.4) | - |  | 0.059 |  |
| Insulin analog | 572 (32.1) | - |  | 303 (28.5) | - |  | 0.050 |  |
| GLP-1 analog | 141 (7.9) | - |  | 62 (5.8) | - |  | 0.039 |  |
| SGLT2-Inhibitor | 130 (7.3) | - |  | 40 (3.8) | - |  | <0.0001 |  |
| **Socio-economic status** | | | | | | | | |
| Marital status |  |  |  |  |  |  |  |  |
| Married | 961 (54.0) | 2896 (53.0) | 0.492 | 615 (58.0) | 2563 (56.0) | 0.232 | 0.034 | 0.003 |
| Single | 347 (19.5) | 1254 (23.0) | 0.002 | 142 (13.4) | 1005 (21.9) | <0.0001 | <0.0001 | 0.237 |
| Divorced | 338 (19.0) | 975 (17.8) | 0.284 | 212 (20.0) | 719 (15.7) | 0.0007 | 0.500 | 0.005 |
| Widowed | 132 (7.4) | 325 (6.0) | 0.028 | 90 (8.5) | 279 (6.1) | 0.005 | 0.298 | 0.755 |
| Educational level |  |  |  |  |  |  |  |  |
| <10 years | 643 (36.1) | 1417 (25.9) | <0.0001 | 375 (35.4) | 1179 (25.8) | <0.0001 | 0.704 | 0.847 |
| 10-12 years | 830 (46.6) | 2560 (46.8) | 0.845 | 478 (45.1) | 1919 (41.9) | 0.060 | 0.443 | <0.0001 |
| >12 years | 289 (16.2) | 1414 (25.9) | <0.0001 | 194 (18.3) | 1431 (31.3) | <0.0001 | 0.153 | <0.0001 |
| Country of birth |  |  |  |  |  |  |  |  |
| Sweden | 1421 (79.7) | 4582 (83.8) | <0.0001 | 867 (81.8) | 3959 (86.5) | <0.0001 | 0.182 | 0.0002 |
| Europe except Sweden | 231 (13.0) | 597 (10.9) | 0.019 | 140 (13.2) | 431 (9.4) | 0.0002 | 0.852 | 0.013 |
| Outside Europe | 130 (7.3) | 286 (5.2) | 0.001 | 53 (5.0) | 187 (4.1) | 0.184 | 0.016 | 0.007 |

Categorical variables are presented as n (%) and continuous variables as mean (SD).

Variables including duration of diabetes, BMI, smoking status, HbA1c, eGFR, cholesterol, triglycerides, albuminuria, and systolic/diastolic blood pressure were retrieved from the National Diabetes Register and were therefore only available for patients with type 2 diabetes.

Abbreviations: No DM: No Diabetes Mellitus, eGFR: Estimated Glomerular Filtration Rate, LBBB: Left Bundle Branch Block, VT/VF: Ventricular Tachycardia/Ventricular Fibrillation, AV Block: Atrioventricular Block, MRA: Mineralocorticoid Receptor Antagonist, ASA: Acetylsalicylic Acid, RAS: Renin-Angiotensin System, PCSK9I: Proprotein Convertase Subtilisin/Kexin Type 9 Inhibitor, GLP-1: Glucagon-Like Peptide-1, SGLT2: Sodium-Glucose Cotransporter-2.

**Table S3- Comparison of indication for de-novo ICD implantation between type 2 diabetes vs. No-DM patients in subgroups receiving ICD for primary and secondary prevention**

|  | Primary prevention | |  | Secondary prevention | |  |
| --- | --- | --- | --- | --- | --- | --- |
|  | Type 2 diabetes  **n= 1782 (24.6%)** | No DM  **n= 5465 (75.4%)** | p-value | Type 2 diabetes  **n= 1060 (18.8%)** | No DM  **n= 4577 (81.2%)** | p-value |
| **Symptoms** | | | | | | |
| Breathlessness/tiredness | 218 (12.2) | 659 (12.1) | 0.844 | - | - |  |
| Asymptomatic | 40(2.1) | 147 (2.7) | 0.224 | 85 (8.0) | 321 (7.0) | 0.254 |
| Heart failure symptoms | 417 (23.4) | 1033 (18.9) | <0.0001 | - | - |  |
| Palpitations | 178 (10.0) | 808 (14.8) | <0.0001 | - | - |  |
| Syncope | 90 (5.1) | 341 (6.2) | 0.065 | 347 (32.7) | 1582 (34.6) | 0.258 |
| Primary profylax | 839 (47.1) | 2477 (45.3) | 0.196 | - | - |  |
| Aborted sudden death | - | - |  | 628 (59.3) | 2674 (58.4) | 0.624 |
| **Etiology** | | | | | | |
| ARVC | 4 (0.2) | 163 (3.0) | <0.0001 | 2 (0.2) | 101 (2.2) | <0.0001 |
| Amyloidosis | 4 (0.2) | 23 (0.4) | 0.237 | 1 (0.1) | 15 (0.3) | 0.335 |
| Other structural heart disease | 42 (2.4) | 217 (4.0) | 0.001 | 47 (4.4) | 367 (8.0) | <0.0001 |
| Idiopathic | 86 (4.8) | 353 (6.5) | 0.012 | 116 (10.9) | 981 (21.4) | <0.0001 |
| Ischemic heart disease | 788 (44.2) | 1718 (31.4) | <0.0001 | 569 (53.7) | 1559 (34.1) | <0.0001 |
| Dilated cardiomyopathy | 353 (19.8) | 1301 (23.8) | 0.0005 | 82 (7.7) | 453 (9.9) | 0.031 |
| Hypertrophic cardiomyopathy | 47 (2.6) | 444 (8.1) | <0.0001 | 23 (2.2) | 172 (3.8) | 0.011 |
| Ischemic cardiomyopathy | 343 (19.3) | 723 (13.2) | <0.0001 | 119 (11.2) | 276 (6.0) | <0.0001 |
| Valvular heart disease | 8 (0.5) | 40 (0.7) | 0.201 | 9 (0.9) | 79 (1.7) | 0.038 |
| Congenital heart disease | 5 (0.3) | 57 (1.0) | 0.002 | 5 (0.5) | 82 (1.8) | 0.002 |
| Long QT syndrome | 1 (0.1) | 81 (1.5) | <0.0001 | 20 (1.9) | 157 (3.4) | 0.009 |
| Myocarditis | 6 (0.3) | 60 (1.1) | 0.003 | 5 (0.5) | 100 (2.2) | 0.0002 |
| Post-infarction | 88 (4.9) | 182 (3.3) | 0.002 | 58 (5.5) | 184 (4.0) | 0.036 |
| Sarcoidosis | 5 (0.3) | 98 (1.8) | <0.0001 | 4 (0.4) | 40 (0.9) | 0.098 |
| Other^*^ | 2 (0.1) | 5 (0.1) | 0.683 | 0 (0.0) | 11 (0.2) | 0.236 |
| **ECG-findings** | | | | | | |
| VF | 15 (0.8) | 64 (1.2) | 0.245 | 435 (41.0) | 1990 (43.5) | 0.148 |
| VT | 237 (13.3) | 1031 (18.9) | <0.0001 | 361 (34.1) | 1573 (34.4) | 0.848 |
| VT+VF | 31 (1.74) | 101 (1.85) | 0.766 | 197 (18.6) | 751 (16.4) | 0.088 |
| NSVT | 68 (3.8) | 284 (5.2) | 0.019 | 53 (5.0) | 219 (4.8) | 0.768 |
| Primary prophylaxis | 1431 (80.3) | 3985 (72.9) | <0.0001 | 14 (1.3) | 42 (0.9) | 0.233 |

Abbreviations: No DM: No Diabetes Mellitus, VT/VF: Ventricular Tachycardia/Ventricular Fibrillation, ARVC: Arrhythmogenic Right Ventricular Cardiomyopathy, NSVT: Non-Sustained Ventricular Tachycardia

*Other included Enodcarditis, High proportion of right ventricular pacing, Cytostatic-induced cardiomyopathy, Post TAVI (Transcatheter Aortic Valve Implantation) and conduction system fibrosis.

**Table S4- Cause-of-death categories based on ICD-10 codes**

|  | Type 2 diabetes | No-DM |
| --- | --- | --- |
| A00-B99 - Certain infectious and parasitic diseases | 79 (4.9) | 122 (3.9) |
| D50-D89 - Diseases of the blood and blood-forming organs and certain disorders involving the immune mechanism | 10 (0.6) | 18 (0.6) |
| E00-E89 - Endocrine, nutritional and metabolic diseases | 123 (7.7) | 57 (1.8) |
| I00-I99 - Diseases of the circulatory system   - I20-I25 Ischemic heart disease - I46, I49.0, I47.2 VT/VF/SCD - I48 Atrial fibrillation and flutter - I50 Heart failure - I60-I69 Cerebrovascular disease | 1128 (70.5)  601 (37.6)  27 (1.7)  37 (2.3)  217 (13.6)  33 (2.1) | 2324 (74.8)  1044 (33.6)  63 (2.0)  89 (2.9)  393 (12.7)  90 (2.9) |
| J00-J99 - Diseases of the respiratory system | 123 (7.7) | 245 (7.9) |
| K00-K95 - Diseases of the digestive system | 35 (2.2) | 98 (3.2) |
| L00-L99 - Diseases of the skin and subcutaneous tissue | 6 (0.4) | 7 (0.2) |
| N00-N99 - Diseases of the genitourinary system | 32 (2.0) | 81 (2.6) |
| R00-R99 - Symptoms, signs and abnormal clinical and laboratory findings, not elsewhere classified | 61 (3.8) | 147 (4.7) |
| V00-Y99 - External causes of morbidity | 3 (0.2) | 6 (0.2) |
| **Total** | **1600** | **3105** |

Abbreviations: No-DM: patients without diabetes, SCD: sudden cardiac death, VF: ventricular fibrillation, VT: ventricular tachycardia.

**Table S5- Risk of MACE and all-cause mortality in type 2 vs. No-DM (reference) patients**

|  | **Hazard Ratio (95% CI)** | p-value | Model 1^a^ | p-value | Model 2^b^ | p-value |
| --- | --- | --- | --- | --- | --- | --- |
| MACE | 1.87 (1.71-2.05) | <0.0001 | 1.54 (1.41-1.69) | <0.0001 | 1.41 (1.28-1.55) | <0.0001 |
| - CV-deaths | 2.19 (1.93-2.48) | <0.0001 | 1.70 (1.50-1.93) | <0.0001 | 1.50 (1.33-1.70) | <0.0001 |
| - Non-fatal MI | 1.94 (1.65-2.27) | <0.0001 | 1.66 (1.41-1.95) | <0.0001 | 1.54 (1.31-1.81) | <0.0001 |
| - Non-fatal stroke | 1.46 (1.23-1.73) | <0.0001 | 1.31 (1.10-1.56) | 0.003 | 1.28 (1.07-1.53) | 0.007 |
| All cause mortality | 1.95 (1.81-2.11) | <0.0001 | 1.53 (1.41-1.67) | <0.0001 | 1.43 (1.32-1.55) | <0.0001 |

Data are presented as hazard ratios, unadjusted and adjusted in two models, with 95% confidence interval (CI).

Adjustment made in following models:

a: Model 1: Adjusted for sex, age, marital status, education, country of birth,

b: Model 2: Adjusted for model 1 and in addition previous ischemic heart disease and heart failure, atrial fibrillation, peripheral artery disease, stroke and chronic obstructive pulmonary disease

Abbreviations: MACE: major adverse cardiovascular event, CI: confidence interval, CV: cardiovascular, MI: myocardial infarction, No-DM: patients without diabetes

**Table S6- Risk of MACE and all-cause mortality in type 2 diabetes vs. No-DM patients (reference) in subgroups receiving ICD for primary and secondary prevention**

1. Primary prophylaxis group:

| Type 2 diabetes vs No-DM | Hazard Ratio (95% CI) | p-value | Model 1 | p-value | Model 2 | p-value |
| --- | --- | --- | --- | --- | --- | --- |
| MACE | 1.66 (1.47-1.87) | <0.0001 | 1.42 (1.25- 1.60) | <0.0001 | 1.31 (1.15-1.48) | <0.0001 |
| - CV-deaths | 1.90 (1.62-2.24) | <0.0001 | 1.58 (1.34-1.86) | <0.0001 | 1.42 (1.20-1.68) | <0.0001 |
| - Non-fatal MI | 1.82 (1.47-2.26) | <0.0001 | 1.60 (1.28-2.00) | <0.0001 | 1.49 (1.19-1.86) | 0.0005 |
| - Non-fatal stroke | 1.25 (0.99-1.58) | 0.066 | 1.13 (0.89-1.44) | 0.320 | 1.09 (0.86-1.39) | 0.479 |
| All cause mortality | 1.73 (1.56-1.92) | <0.0001 | 1.44 (1.30-1.60) | <0.0001 | 1.34 (1.21-1.49) | <0.0001 |

1. Secondary prophylaxis group:

| Type 2 diabetes vs No-DM | Hazard Ratio (95% CI) | p-value | Model 1 | p-value | Model 2 | p-value |
| --- | --- | --- | --- | --- | --- | --- |
| MACE | 2.22 (1.93-2.54) | <0.0001 | 1.74 (1.51-2.01) | <0.0001 | 1.54 (1.34-1.78) | <0.0001 |
| - CV-deaths | 2.64 (2.18-3.19) | <0.0001 | 1.88 (1.55- 2.28) | <0.0001 | 1.62 (1.33-1.97) | <0.0001 |
| - Non-fatal MI | 2.15 (1.70-2.72) | <0.0001 | 1.80 (1.41-2.29) | <0.0001 | 1.60 (1.26-2.03) | 0.0001 |
| - Non-fatal stroke | 1.80 (1.40-2.31) | <0.0001 | 1.59 (1.23-2.06) | 0.0004 | 1.52 (1.17-1.97) | 0.002 |
| All cause mortality | 2.30 (2.04-2.60) | <0.0001 | 1.66 (1.47-1.88) | <0.0001 | 1.55 (1.37-1.76) | <0.0001 |

Data are presented as hazard ratios, unadjusted and adjusted in two models, with 95% confidence interval (CI)

Adjustment made in following models:

a: Model 1: sex, age, marital status, education, country of birth,

b: Model 2: Adjusted for model 1 and in addition previous ischemic heart disease and heart failure, atrial fibrillation, peripheral artery disease, stroke and chronic obstructive pulmonary disease

Abbreviations: MACE: major adverse cardiovascular event, CI: confidence interval, CV: cardiovascular, MI: myocardial infarction, No-DM: patients without diabetes

**Table S7- Risk of MACE and all-cause mortality in patients with primary vs. secondary (reference) prophylactic ICD based on diabetes status**

1. Type 2 diabetes group

| Primary vs secondary | Hazard Ratio (95% CI) | p-value | Model 1 | p-value | Model 2 | p-value |
| --- | --- | --- | --- | --- | --- | --- |
| MACE | 0.78 (0.67- 0.91) | 0.001 | 0.83 (0.71-0.97) | 0.017 | 0.75 (0.63-0.89) | 0.001 |
| - CV-deaths | 0.84 (0.69-1.03) | 0.099 | 0.99 (0.81-1.22) | 0.928 | 0.77 (0.61-0.97) | 0.028 |
| - Non-fatal MI | 0.75 (0.58-0.97) | 0.026 | 0.74 (0.57-0.96) | 0.024 | 0.81 (0.60-1.09) | 0.164 |
| - Non-fatal stroke | 0.69 (0.52-0.93) | 0.013 | 0.69 (0.51-0.93) | 0.014 | 0.68 (0.49-0.94) | 0.021 |
| All cause mortality | 0.88 (0.77 -0.99) | 0.046 | 1.04 (0.91-1.18) | 0.617 | 0.79 (0.68-0.91) | 0.002 |

1. No-DM group

| Primary vs secondary | Hazard Ratio (95% CI) | p-value | Model 1 | p-value | Model 2 | p-value |
| --- | --- | --- | --- | --- | --- | --- |
| MACE | 1.06 (0.95-1.17) | 0.297 | 1.06 (0.96-1.18) | 0.248 | 0.91 (0.81- 1.02) | 0.100 |
| - CV-deaths | 1.19 (1.03-1.38) | 0.021 | 1.27 (1.09-1.47) | 0.002 | 0.88 (0.74-1.03) | 0.115 |
| - Non-fatal MI | 0.90 (0.74-1.08) | 0.241 | 0.89 (0.74-1.07) | 0.210 | 0.88 (0.72-1.08) | 0.224 |
| - Non-fatal stroke | 0.98 (0.82-1.18) | 0.858 | 0.98 (0.81-1.17) | 0.795 | 0.93 (0.76-1.13) | 0.459 |
| All cause mortality | 1.18 (1.08-1.29) | 0.0003 | 1.24 (1.13-1.36) | <0.0001 | 0.95 (0.86-1.05) | 0.334 |

Data are presented as hazard ratios, unadjusted and adjusted in two models, with 95% confidence interval (CI).

Adjustment made in following models:

a: Model 1: Adjusted for sex, age, marital status, education, country of birth,

b: Model 2: Adjusted for model 1 and in addition previous ischemic heart disease and heart failure, atrial fibrillation, peripheral artery disease, stroke and chronic obstructive pulmonary disease

Abbreviations: MACE: major adverse cardiovascular event, CI: confidence interval, CV: cardiovascular, MI: myocardial infarction, No-DM: patients without diabetes

**Table S8- Absolute event rates (events per 100 person-years) for MACE and mortality by type 2 diabetes status and prevention type**

|  |  | MACE | All-cause mortality |
| --- | --- | --- | --- |
| Type 2 diabetes | Primary prevention | 4.79 | 6.66 |
|  | Secondary prevention | 5.92 | 7.68 |
| No-DM | Primary prevention | 2.78 | 3.95 |
|  | Secondary prevention | 2.59 | 3.38 |

Abbreviations: MACE: major adverse cardiovascular event, No-DM: patients without diabetes

# **Definitions of variables**

Estimated glomerular filtration rate, eGFR (mL/min/1.73 m^2^) was derived using the CKD-EPI equation.

Glycated hemoglobin (HbA1c) levels were expressed in both mmol/mol and percentage, according to the standards set by the International Federation of Clinical Chemistry and Laboratory Medicine (IFCC) and the Diabetes Control and Complications Trial (DCCT).

Microalbuminuria was defined by at least two positive test results within one year, with an albumin-to-creatinine ratio of 3–30 mg/mmol (30–300 mg/g) or a urinary albumin excretion rate of 20–200 µg/min (20–300 mg/L).

Macroalbuminuria was defined as an albumin-to-creatinine ratio exceeding 30 mg/mmol (approximately 300 mg/g or more) or a urinary albumin excretion rate over 200 µg/min (more than 300 mg/L). Estimated Glomerular Filtration Rate (eGFR) was derived from serum creatinine levels using the Chronic Kidney Disease Epidemiology Collaboration (CKD-EPI) equation.

A smoker was classified as an individual who smoked at least one cigarette daily, smoked a pipe daily, or had quit smoking within the previous three months.

Body Mass Index (BMI) was calculated from weight and height data obtained from primary care units and hospital outpatient clinics.

Blood pressure (BP) measurements were taken as the average of two readings (Korotkoff phases 1–5) with the patient seated or lying down for 2 min, using a cuff of the appropriate size.

High-density lipoprotein (HDL) and low-density lipoprotein (LDL) cholesterol levels were measured in mmol/L.

# **Classification of prevention types from Swedish ICD and Pacemaker Registry**

ICD prevention types (primary or secondary) were defined as follows based on symptoms and ECG findings registered in the Swedish ICD and Pacemaker register:

**Primary prophylaxis:**

Symptom: Asymptomatic VT/VF; NSVT marked under ECG-findings = primary.

Symptom: Syncope; marked as primary under ECG-findings = primary.

Symptom: Dizziness, shortness of breath/fatigue, heart failure, palpitations = primary prophylaxis regardless of what is marked under ECG-findings.

**Secondary prophylaxis:**

Symptom: "Survived sudden death" = always secondary.

Symptom: "Syncope" = always secondary unless marked as primary under ECG-findings, in which case it is primary.

Symptom: Asymptomatic VT/VF + marked as ventricular tachycardia under ECG-findings = secondary.
